# On the Superconductivity Suppression in Eu_1__−_*_x_*Pr*_x_*Ba_2_Cu_3_O_7__−_*_δ_*

**DOI:** 10.3390/ma14133503

**Published:** 2021-06-23

**Authors:** Paweł Pęczkowski, Piotr Zachariasz, Cezariusz Jastrzębski, Jarosław Piętosa, Elżbieta Drzymała, Łukasz Gondek

**Affiliations:** 1 Institute of Physical Sciences, Faculty of Mathematics and Natural Sciences, School of Exact Sciences, Cardinal Stefan Wyszyński University, K. Wóycickiego 1/3 Street, 01-938 Warsaw, Poland; 2 LTCC Technology and Printed Electronics Research Group, Łukasiewicz Research Network—Institute of Microelectronics and Photonics, Zabłocie 39 Street, 30-701 Kraków, Poland; piotr.zachariasz@imif.lukasiewicz.gov.pl; 3 Faculty of Physics, Warsaw University of Technology, Koszykowa 75 Street, 00-662 Warsaw, Poland; cezjas@gmail.com; 4 Group of Phase Transition, Division of Physics of Magnetism, Institute of Physics, Polish Academy of Sciences, Lotników 32/46 Avenue, 02-668 Warsaw, Poland; pietosa@ifpan.edu.pl; 5 Department for Functional Nanomaterials, The Henryk Niewodniczański Institute of Nuclear Physics, Polish Academy of Sciences, W.E. Radzikowskiego 152 Street, 31-342 Kraków, Poland; elzbieta.drzymala@ifj.edu.pl; 6 Department of Solid State Physics, Faculty of Physics and Applied Computer Science, AGH University of Science and Technology, A. Mickiewicza 30 Avenue, 30-059 Kraków, Poland; lgondek@agh.edu.pl

**Keywords:** high-temperature superconductors, solid-state reaction method, magnetism, X-ray diffraction, Raman spectroscopy, X-ray absorption spectroscopy

## Abstract

This article reports on the non-trivial suppression of superconductivity in the Eu_1−*x*_Pr*_x_*BCO cuprates. As non-magnetic Eu^3+^ ions are replaced by Pr^3+^ carrying a magnetic moment, spin-related superconductivity loss can be expected. The research shows that the superconductivity disappearance for *x* > 0.4 results from depletion of the carriers and their localization. The above conclusion was drawn by low-temperature X-ray diffraction analysis showing increased characteristic phonon frequencies with Pr content. This mechanism should promote electron–phonon coupling, at least for acoustic phonons. However, the inverse phenomenon was detected. Namely, there is a gradual deterioration of the optical phonons responsible for vibration of the Cu–O bonds with Pr increasing, as evidenced by Raman spectroscopy. Furthermore, the results of X-ray absorption spectroscopy precisely showed the location of the carriers for Pr-rich specimens. Finally, a schematic diagram for Eu_1−*x*_Pr*_x_*BCO is proposed to consolidate the presented research.

## 1. Introduction

In 1986, Bednorz and Müller discovered the high-temperature superconductivity (HTS) in La–Ba–Cu–O, and the following year, they received the Nobel Prize in Physics, which indicates the incredible significance of this discovery for the energy and space industries, as well as for numerous technical and medical applications. The last point concerns, i.e., the superconducting magnets, SQUID-based devices, superconducting JJ-arrays (Josephson junctions), fault-current limiters, or magnetic levitation transport systems. Finally, the possibility to measure weak magnetic fields has released a wide range of biomedical applications (e.g., magnetoencephalography).

Historically, La–Ba–Cu–O was the first high-temperature superconductor (HTS) with a critical temperature (*T*_c_) close to 36 K [1]. Later, by applying hydrostatic pressure, Chu [2] increased the superconducting state of La_5__−_*_x_*Ba*_x_*Cu_5_O_5(3__−_*_y_*_)_ up to 50 K, showing in practice that various conditions can effectively affect *T*_c_.

In turn, a significant rise in critical temperature was observed for the REBa_2_Cu_3_O_7__−_*_δ_* family (RE—rare earth element); e.g., Wu and Chu [3] proved the oxygen-dependent *T*_c_ ≈ 90 K for YBa_2_Cu_3_O_7__−_*_δ_*. Further studies of other rare earth elements [4,5,6,7,8,9,10,11] show that *T*_c_ can be even above 90 K for most RE^3+^ ions, despite possessing a spin magnetic moment, which is typically antagonistic for the superconducting state.

Nevertheless, there are a few exclusions to this rule. CeBa_2_Cu_3_O_7__−_*_δ_* and TbBa_2_Cu_3_O_7__−_*_δ_* [12] principally do not crystalize in the superconducting orthorhombic structure (*Pmmm*, No. 47; so-called RE-123 phase). In turn, PrBa_2_Cu_3_O_7−*δ*_ crystallizes in the orthorhombic Pr-123 structure; however, the superconductivity of bulk samples is rarely reported, which is presumably due to the Pr/Ba mixing being difficult to avoid [13,14,15].

As predicted theoretically, PrBa_2_Cu_3_O_7−*δ*_ shows superconductivity at *T*_c_ ≈ 90 K with 90% ÷ 10% transition width of Δ*T* ≈ 20 K or less [16]. This behavior has been confirmed by studies on the superconductivity of thin layers [17], single crystals [18,19,20], and excellent polycrystalline samples [21]. However, the latter materials are challenging to synthesize since Pr-based ceramics are sensitive to sintering conditions, promoting the Pr/Ba exchange in the crystal structure [15,21]. Furthermore, since the interplanar bonds in cuprates are mainly ionic, the difference in radii Pr^3+^ (113.0 pm) and Ba^2+^ (149.0 pm) impacts the *c* lattice parameter of the *Pmmm* structure. In general, the *c* lattice constant for non-superconducting material is smaller than for the superconducting one [21].

Many researchers investigated the Y_1−*x*_Pr*_x_*Ba_2_Cu_3_O_7__−_*_δ_* [22,23,24] and Gd_1−*x*_Pr*_x_*Ba_2_Cu_3_O_7__−_*_δ_* systems [25] and found that the critical temperature (*T*_c_) decreased monotonically with Pr concentration, similar to YBa_2_Cu_3_O_7__−_*_δ_*–YMnO_3_ composites enriched with YMnO_3_ [26].

NdBa_2_Cu_3_O_7__−_*_δ_* is a familiar system where Ba^2+^ can substitute Nd^3+^. However, the formation of Nd–Ba defects is much less frequent than the development of Pr–Ba defects [27]. The *T*_c_ variability can be explained by the Nd/Ba mixing frequency controlling the charge transfer from Cu–O_2_ planes to Cu–O chains [27].

Research on the Eu_1−*x*_Pr*_x_*Ba_2_Cu_3_O_7__−_*_δ_* system (regularly called (Eu,Pr)BCO or (Eu,Pr)-123 phase) was initiated in 1990, focusing on Mössbauer ^151^Eu spectroscopy and superconductivity suppression by Pr^4+^ ions at *x* = 0.4 [28]. In addition, research on specific heat has also been reported [29], where Pr contribution in the metallic phase is manifested by a broad anomaly quite well described by the Kondo method. Then, the entropy correlated with Pr anomalies in metallic and insulating phases indicates a twofold ground state of Pr with a valence close to 4^+^ in the crystalline electric field.

This paper aims to explain the non-trivial suppression of superconductivity in the (Eu_1__−_*_x_*Pr*_x_*)BCO system (*x* = 0.0; 0.2; 0.4; 0.6; 0.8; 1.0). For the Eu^3+^ ion (4*f* ^6^5*s*^2^5*p*^6^), the orbital and spin contributions in the total angular momentum (*J*) cancel each other, making the Eu-123 phase unique (*J* = 0). On the other hand, the Pr^3+^ ion (4*f* ^2^5*s*^2^5*p*^6^) reveals *J* = 4. However, it is widely accepted that the ground state of the Pr^3+^ multiplet is an intrinsically non-magnetic singlet in a crystal electric field. Thus, superconductivity suppression in this particular case does not seem to be biased by magnetism.

## 2. Materials and Methods

### 2.1. Preparation of (Eu,Pr)BCO by Solid-State Reaction Method

Eu_2_O_3_, Pr_2_O_3_ (Sigma-Aldrich, St. Louis, MO, USA, 99.99% purity), CuO (Alfa-Aesar, Haverhill, MA, USA, 99.999% purity), and BaCO_3_ (POCH, Gliwice, Poland, 99.6% purity) were used to prepare the Eu_1__−_*_x_*Pr*_x_*BCO samples. First, the materials were synthesized by a conventional solid-state reaction method by calcining the stoichiometric weighed substrates at 950 °C for 24 h. Then, the sinters were crushed, ground, and calcination was repeated. Next, sintered materials were ground again and pelletized under a uniaxial pressure of 800 MPa. Finally, the pellets were annealed in an oxygen flow of 20 L/h at 920 °C. After 33 h, the temperature was lowered to 400 °C and held for the next 33 h, as described [8,9,30].

### 2.2. Characterization of Materials

Scanning Electron Microscopy (SEM) with Energy-Dispersive X-Ray Spectroscopy (EDS) was performed on the TESCAN VEGA 3 SBH instrument. The local microstructures were imaged using the SE detector in a high vacuum mode and at a voltage of 30 kV. The EDS detector equipped with the Bruker Esprit software analyzed the chemical compositions of (Eu,Pr)BCO. The SEM/EDS studies were conducted on fractured as well as on polished surfaces of pellets.

X-Ray Diffraction (XRD) studies were performed using the Empyrean PANalytical instrument operating in the Bragg–Brentano geometry with Cu *K*_α_ radiation (*λ* = 1.5406 Å) and NIST LaB_6_ standard for correcting the instrument broadening. Diffractograms were collected over an angular range of 5° < 2*θ* < 130°. Low-temperature studies (15 ÷ 300 K) were made using Oxford Instruments. The specimen position was corrected against thermal displacements. The Rietveld analysis was employed [31,32] to quantify the diffraction patterns of (Eu,Pr)BCO.

Raman vibrational spectra were measured with the Renishaw micro-Raman inVia Reflex instrument in backscattering geometry. A 514 nm line of an Ar-ion laser with a spot diameter of about 1.5 µm was used to excite the material. The laser power has been adjusted so that the cumulative energy dose per sample did not exceed 1 mW to avoid laser-induced degradation effects. Raman spectra were collected cumulatively over three passes each for 30 s to enhance the signal-to-noise ratio.

X-ray absorption spectroscopy (XAS) was employed to inspect the (Eu,Pr)BCO electronic structure. The XAS data were recorded in a total-electron-yield mode from the area of 0.5 × 0.3 mm^2^ with a spectral resolution Δ*E*/*E* of 2.5·10^−4^ and normalized for background calibration measurements. The research was carried out at the SOLARIS National Synchrotron Radiation Centre (Kraków, Poland) on the PEEM/XAS line. The experiment was accomplished in cooperation with the SOLARIS Staff.

Magnetic measurements as a function of temperature (5 ÷ 120 K) and magnetic field up to 50 kOe were carried out with a superconducting quantum interference magnetometer (Quantum Design, San Diego, CA, USA, MPMS-5).

The method of fixed-point titration with sodium thiosulfate (Na_2_S_2_O_3_) was utilized to determine the oxygen concentration [33].

## 3. Results

### 3.1. Microstructural Analysis

**Figure 1:**
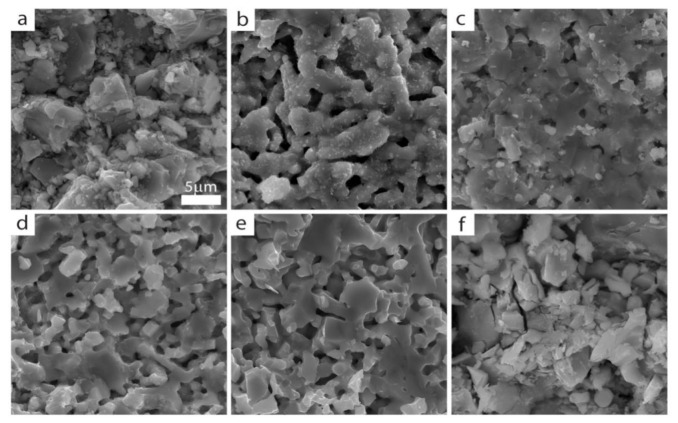
Scanning electron microscopy (SEM) images of the local microstructures: (**a**) EuBCO, (**b**) Eu_0.2_Pr_0.8_BCO, (**c**) Eu_0.4_Pr_0.6_BCO, (**d**) Eu_0.6_Pr_0.4_BCO, (**e**) Eu_0.8_Pr_0.2_BCO, and (**f**) PrBCO.

The local microstructure evolution of the (Eu,Pr)BCO system is presented in Figure 1.

Comparable morphologies can be observed for all Eu_1−*x*_Pr*_x_*BCO (Figure 1b–e), which are essentially different from the parent EuBCO and PrBCO. The latter compounds are characterized by distinct grains with sharp edges and sizes from 5 to 10 μm (for PrBCO even up to 15 μm).

In contrast, the (Eu,Pr)BCO crystallites are finer and show more rounded edges, and it could even be said that the local microstructures of (Eu,Pr)BCO are slightly glassy. The grain sizes rarely exceed 5 μm, and for Eu_0.2_Pr_0.8_BCO (Figure 1b), one can find the delicate crystallites that decorate the surfaces of larger ones. In turn, Eu_0.4_Pr_0.6_BCO (Figure 1c) has the most compact local microstructure of the other compounds.

**Figure 2:**
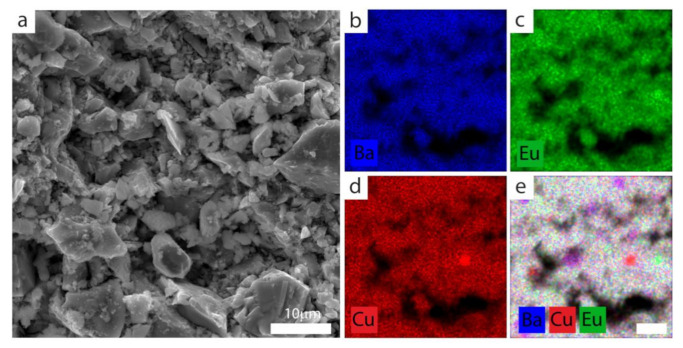
(**a**) Local microstructure (SEM) of EuBCO with EDS elemental maps showing the distribution of (**b**) Ba; (**c**) Eu; (**d**) Cu; (**e**) their overlapping.

Energy-dispersive X-ray spectroscopy was also employed to evaluate the contribution of individual elements (EDS mapping) in the (Eu,Pr)BCO system (Figure 2).

Note that the EDS technique is a local estimation method and does not provide the average information from a bulk sample. However, as shown in Figure 2b–d, uniformly distributed elements in local microstructures develop small inclusion aggregates (Figure 2e).

Taking into account the constituent atoms—barium (blue), europium (green), copper (red)—and the RGB color model, one can estimate the chemical composition of the colored clusters detected by the EDS mapping technique. Then, the magenta aggregates (Figure 2e) should be attributed to the BaCuO_2_ inclusions, the small greenish spots mark the Eu_2_BaCuO_5_ (Eu-211) parasitic phase, and red clusters understandably represent the residual CuO phase. Other (Eu,Pr)BCO exhibit similar behavior, meaning that all specimens possess small although detectable inclusions.

**Figure 3:**
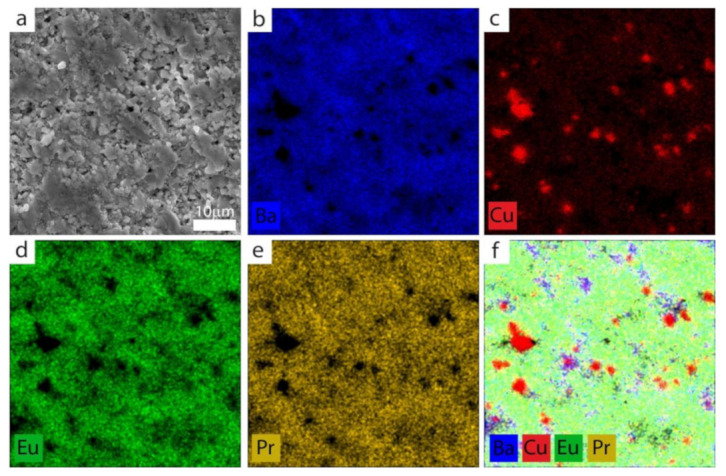
(**a**) Local microstructure (SEM) of Eu_0.6_Pr_0.4_BCO with the corresponding EDS elemental maps showing the distribution of (**b**) Ba; (**c**) Cu; (**d**) Eu; (**e**) Pr; and (**f**) their overlapping.

Mainly red inclusions are visible for (Eu,Pr)BCO, indicating the CuO phase (Figure 3c). After overlapping the maps of the individual elements, the blue precipitations were found (Figure 3f). The specimens mentioned above show neither europium nor praseodymium clusters. As seen in the EDS maps for Eu_0.6_Pr_0.4_BCO (Figure 3d,e), the Pr and Eu elements are homogeneously distributed. In order to estimate the stoichiometry on the macro scale, several regions of interest (ROIs) of 60 × 60 µm^2^ were discriminated for each specimen, and the EDS mapping results are averaged and listed in Table 1.

During the synthesis of (Eu,Pr)BCO materials, the main concerns were maintaining the correct Pr/Eu ratio and the homogeneity of Pr and Eu. Indeed, EDS studies revealed that the Pr/Eu ratio is preserved in specimens. Moreover, relevant ROIs analysis completed on polished specimens (not shown here) with the assumed uncertainty level yielded the exact stoichiometry of EDS mapping.

### 3.2. X-ray Diffraction Analysis

**Figure 4:**
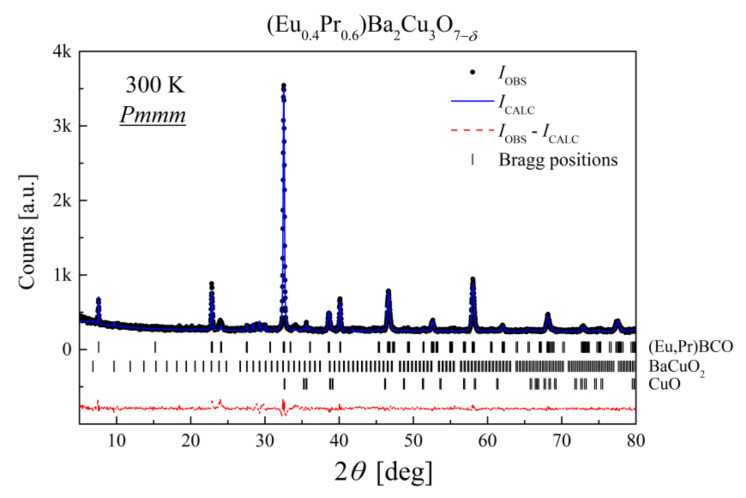
X-ray diffraction pattern of Eu_0.4_Pr_0.6_BCO. The isolated phases (Eu,Pr)BCO (*Pmmm*), BaCuO_2_, and CuO marked with bars are shown in descending order of their contributions.

**Figure 5:**
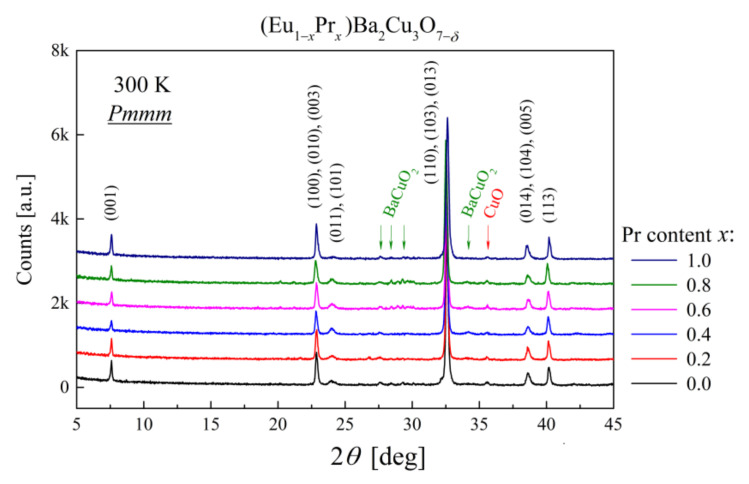
Comparison of the X-ray diffraction patterns for (Eu,Pr)BCO. The parasitic phases are marked with arrows.

According to structural refinement data (Table 2), the dominant phase in (Eu,Pr)BCO was indexed as the orthorhombic *Pmmm* space group. An exemplary diffraction pattern for Eu_0.4_Pr_0.6_BCO with Rietveld analysis is presented in Figure 4. The X-ray data refinement is of good quality with impurity phases less than 14 wt% of the sample. A similar level of impurities was detected for Eu_0.2_Pr_0.8_BCO, while for other composites, the total contribution of parasitic phases was below 5 wt%. A comparison of the collected X-ray patterns for (Eu,Pr)BCO is presented in Figure 5.

**Figure 6:**
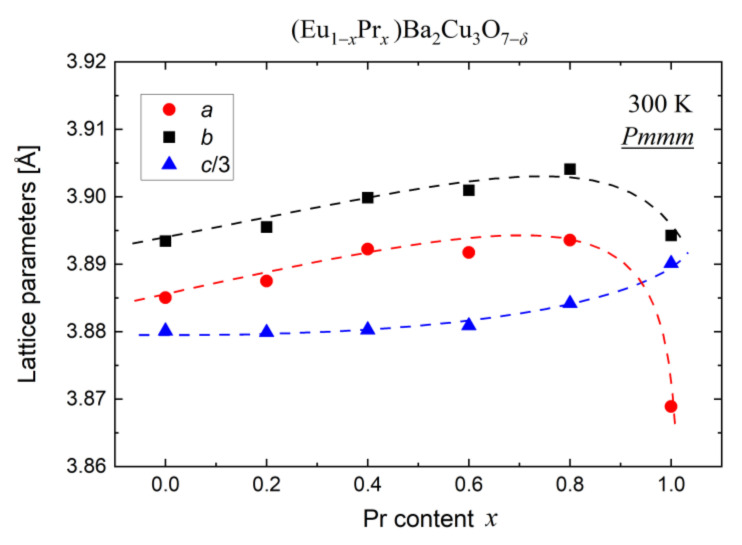
The lattice parameters of (Eu,Pr)BCO distilled at room temperature.

As shown in Figure 5, the PrBCO diffraction pattern exhibits some shifts of the Bragg reflections toward the lower 2*θ* angles compared to other samples. Indeed, analysis of the lattice parameters *a* and *b* suggests that PrBCO is the most oxygen-deficient material due to a significantly reduced unit cell volume. As other (Eu,Pr)BCO materials reveal a regular rise in lattice parameters (Figure 6) due to the lanthanide contraction principle [34,35], it would be expected that substituting Pr for Eu should increase the Pmmm unit cell volume. This effect is directly observed, although only up to *x* ≤ 0.8.

On the other hand, the oxygen impact on the *Pmmm* unit cell size is also known. In the orthorhombic phase, the parameters *a* and *c* decrease with increasing oxygen content [36]. Considering both effects, the rather monotonic growth in lattice parameters (Figure 6) indicates a similar oxygen content for all compounds except for PrBCO. Interestingly, the (Eu,Pr)BCO composition has almost no effect on the lattice parameter (*c*) to *x* ≤ 0.6, reflecting the high structural anisotropy of these compounds.

As the ionic and covalent radii of Pr^3+^ are greater than Eu^3+^, Eu_0.4_Pr_0.6_BCO should be expected to have smaller lattice parameters than PrBCO. However, the unit cell volume is smaller for the latter compound, which is attributed to the oxygen deficiency in PrBCO and/or a change in bonding nature from covalent to more ionic.

**Figure 7:**
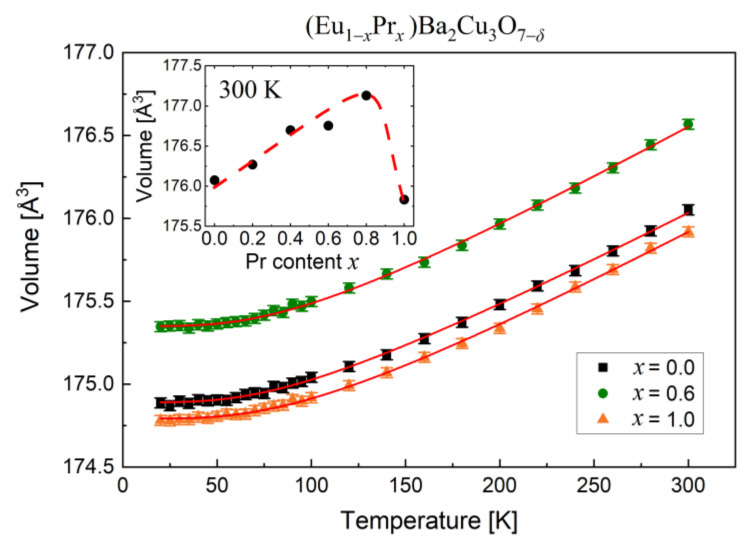
Unit cell volumes of selected (Eu,Pr)BCO as a function of temperature. The solid lines denote the Debye formula as described in the text. The inset shows the volumes of (Eu,Pr)BCO versus Pr content at 300 K.

Thus, detailed structural considerations confirm the BaCuO_2_ and CuO inclusions, while the question of accurately estimating the oxygen in the samples remains. Low-temperature measurements were also carried out to investigate the lattice parameters under and above the superconductivity transition (Figure 7). The results for three selected samples *x* = 0.0, 0.6, and 1.0 reveal significant differences in the volume of unit cells depending on the Pr concentration. These relationships can be approximated by the Debye temperature formula [37]:

(1) $$V=V0+I_{C}\frac{T^{4}}{\theta_{D}^{3}}\int_{0}^{\frac{\theta_{D}}{T}}\frac{x^{3}}{e^{x}-1}dx$$

where *V*_0_ is the unit cell volume at 0 K, *I_C_* is a coefficient including the Grüneisen compressibility parameter, and *θ_D_* is the Debye temperature.

In general, the unit cell volumes increase with temperature (Figure 7), as shown in Equation (1). However, above 180 K, the volumes depend almost linearly on the temperature, with *I*_C_ being the slope. The estimated parameters of Equation (1) are listed in Table 3. The inset in Figure 7 is presenting the change in unit cell volume at ambient temperature as a function of Pr concentration. This dependence clearly shows that the unit cell volume of Eu_0.4_Pr_0.6_BCO is much larger than EuBCO or PrBCO.

A significant rise in Debye temperature as the molar mass of (Eu,Pr)BCO decreases (Table 3) is evidenced by data fitting with Equation (1) and is consistent with the elastic lattice model [38]. In the literature, *θ*_D_ for EuBCO was estimated between 300 and 400 K [39], around 300 K [40], or in the range of 280 ÷ 320 K [41]. On the other hand, for YBCO doped with Pr, the Debye temperature of 350 ÷ 370 K was recorded [42]. Therefore, estimation of the *θ*_D_ parameter in this work correlates with previously reported results.

One can conclude that Pr concentration does not affect the *I*_C_ parameter, which indicates a similar nature of vibrations in the (Eu,Pr)BCO lattice. Thus, variation in the bondings, as mentioned previously, seems unlikely but still possible.

**Figure 8:**
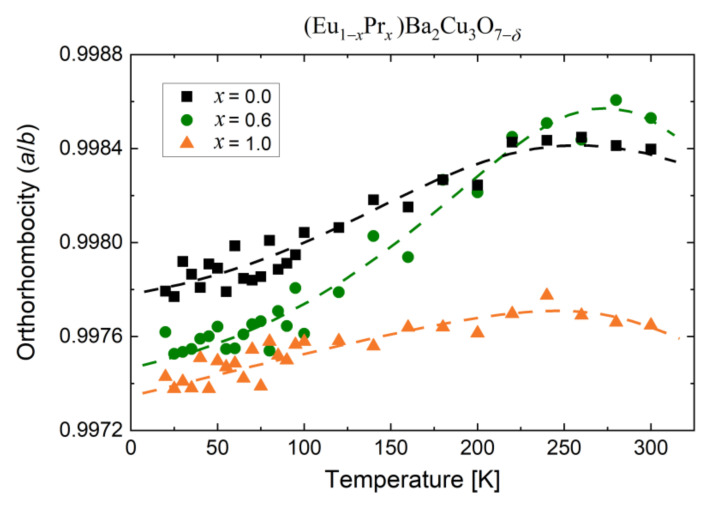
The *Pmmm* unit cell deformation in the basal *a*/*b* plane versus temperature.

Moreover, a distinct difference in the *a*/*b* ratio was observed in the investigated temperature range (Figure 8), with no structural transitions evidenced. The *a*/*b* ratio (orthorhombicity) is a deformation measure in the basal Cu–O_1−_*_δ_* planes; i.e., the crystallographic planes with extra O atoms adopted to *Pmmm* structure during the air-assisted annealing process.

There is a clear tendency for the *a*/*b* ratio o increase with temperature; however, the magnitude of these changes is different. The rate of orthorhombicity change for PrBCO is slowest with temperature, while the *a*/*b* rate is slightly higher for EuBCO. The found difference indicates the high rigidity of the crystallographic structures for parent compounds. Nevertheless, the most interesting behavior is observed for Eu_0.4_Pr_0.6_BCO, consisting of significant growth in the *a*/*b* ratio. At low temperature, the orthorhombicity is closer to PrBCO, while above 200 K, the *a*/*b* ratio is even higher than for EuBCO. It suggests a relatively large susceptibility of the lattice constant (*a*) to temperature changes, in contrast to the lattice parameter (*b*).

### 3.3. Raman Spectroscopy

Two remarks on Raman-scattering spectroscopy for REBCO should be mentioned [43,44,45]. First, the more free charge carriers are in the material, the higher attenuation of the Raman signal is observed. Therefore, large bandgap insulators have more pronounced phonon modes in the spectrum as compared to metals.

Second, for HTS superconductors, there is a relatively high density of carriers in the material and high absorption of visible light by the sample. Therefore, extracting the sophisticated information from Raman spectra requires low laser power densities of the excitation beam, which unfortunately provides a poor signal-to-noise ratio in the Raman spectra; however, it avoids thermal effects and specimen degradation processes.

The standard YBCO spectrum composes of three separated regions of vibrational bands [46]. Low-frequency phonon modes at ~120 cm^−1^ and ~145 cm^−1^ are attributed to vibrations of Ba and Cu(2) atoms, respectively. The following two bands, O(2) – O(3) (out-of-phase) and O(2) + O(3) (in-phase) [47] located around 330 cm^−1^ and 440 cm^−1^, are directly related to the vibrations of oxygen atoms associated with conducting Cu(2)–O_2_ planes.

**Figure 9:**
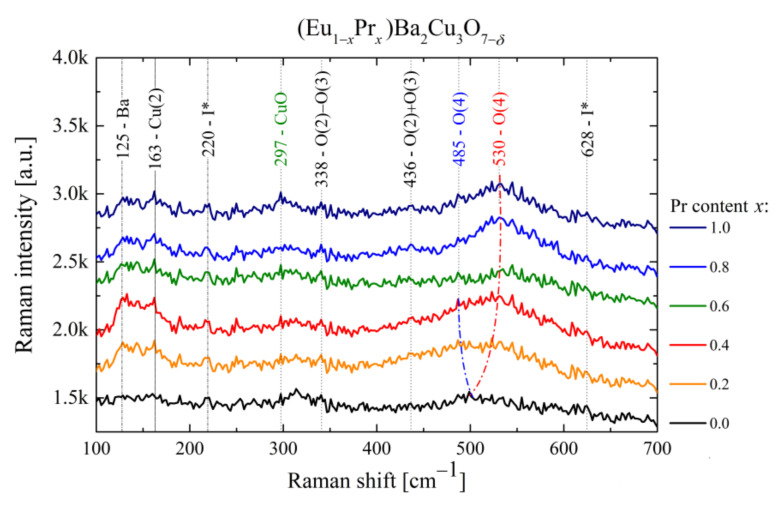
The Raman scattering vibrational spectra for (Eu_,_Pr)BCO as a function of Pr content. I* denotes the BaCuO_2_ inclusions (220 cm^−1^) and cation disorders (628 cm^−1^), possibly indicating the structural defects in Cu–O chains [51,52].

In turn, the O(4) oxygen band detected at 500 cm^−1^ is the most reliable indicator of the YBCO superconductivity, and its position is strongly correlated to the sample heat treatment. Furthermore, the location of this peak is related to an oxygen atom referring to partially occupied Cu–O_1−_*_δ_* planes of an insulating character, which are responsible for the oxygen deficiency in REBa_2_Cu_3_O_7−_*_δ_* materials. Most importantly, for oxygen-rich REBCO (*δ* ≤ 0.35) with a well*-*defined *Pmmm* orthorhombic symmetry, the O(4) phonon mode is meaningly shifted below 500 cm^−1^, while the position of the O(4) peak above 500 cm^−1^ is characteristic for the non-superconducting *P*4*mm* tetragonal structure [48]. Hence, the Raman spectra of (Eu_,_Pr)BCO clearly show the destructive nature of the PrBCO component (Figure 9).

The most characteristic phonon mode at 500 cm^−1^ splits into two bands. The upper-frequency band O(4) shifts up to 530 cm^−1^, whereas the lower frequency band O(4) is shifted to 485 cm^−1^ with a smaller and monotonically disappearing intensity peak compared to the 530 cm^−1^ one. It is established that the Raman frequency of the O(4) peak decreases from 500 to 485 cm^−1^ when REBCO_7−_*_δ_* statistically loses about one oxygen atom (*δ* ≈ 1). Thus, one should expect a decrease in the oxygen stoichiometric index to a value for which (Eu,Pr)BCO_7−_*_δ_* is no longer superconductor. For parent EuBCO and PrBCO compounds, the critical oxygen indexes were reported to be 6.5 and 6.6, respectively [49,50].

### 3.4. X-ray Absorption Spectroscopy

**Figure 10:**
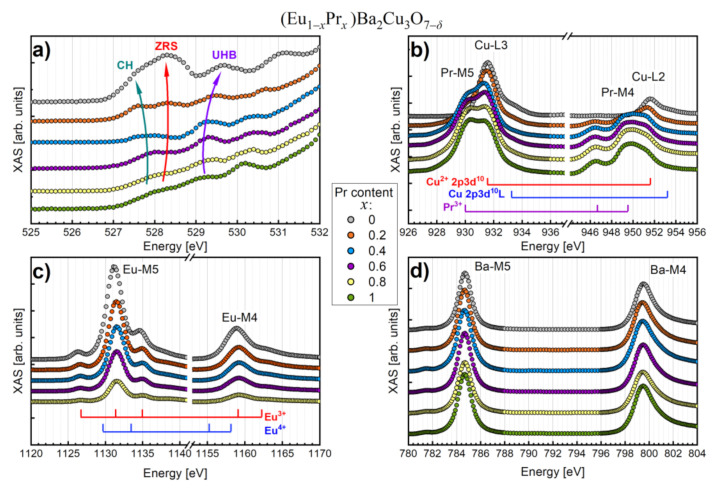
XAS spectra for (Eu,Pr)BCO system: (**a**) O K-edge, (**b**) Cu L2,3-edges with Pr M4,5-edges, (**c**) Eu M4,5-edges, and (**d**) Ba M4,5-edges. The valence states of Eu, Pr, and Cu, as well as the Cu-O chain holes (CH), Zhang–Rice singlets (ZRS), and upper Hubbard band (UHB) states, are marked.

X-ray absorption spectroscopy (XAS) is a proper in situ technique to analyze materials for the electronic structure, local bonding environments, or oxidation states. For example, Figure 10a shows the XAS spectra for the O K-edge (1*s* → 2*p*).

Considering that the 2*p* electron levels of oxygen are hybridized with the 3*d* Cu orbitals, data inspection provides crucial information on the electric charge doping of the Cu–O_2_ planes [53,54,55,56,57]. The XAS spectra (Figure 10a) are characterized by three significant contributions originating from the chain holes (CH) at 527.6 eV, the Zhang–Rice singlets (ZRS) of the hybridized Cu 3*d_x_*^2^_−_*_y_*^2^ and O 2*p_xy_* orbitals within Cu–O_2_ planes, as well as the upper Hubbard band (UHB) mainly related to the conduction band.

Furthermore, it is worth noting that the relative contributions of the ZRS and UHB states are related to the doping levels of the Cu–O_2_ planes [53,54,55,56]. In other words, the ratio of ZRS to UHB is a straightforward indicator of the superconducting properties of cuprates. Hence, the ZRS peak is dominant for the Pr content of *x* ≤ 0.4, while the UHB contribution is far more pronounced for *x* > 0.4.

Moreover, theory predicts a shift of the ZRS peak toward lower energies while moving the UHB component toward higher energies as material enters the superconducting regime [53]. The above effects were also evidenced experimentally in the Y_1−*x*_Ca*_x_*Ba_2_Cu_3_O_7−_*_δ_* system [56]. The CH and ZRS states must also be reflected in the Cu L-edge due to a screening effect of the so-called ligand hole (L) on the O 2*p* orbital. Since this kind of screening occurs for the square Cu–O_2_ planes and straight Cu–O chains, both CH and ZRS effects should contribute to the Cu 2*p*3*d*^10^L final state.

A partial overlapping with the Pr M4,5-edges complicates data analysis of the Cu L2,3-edges (Figure 10b). Nevertheless, the most relevant information can still be extracted. The final state with a hole on the ligand can be recognized as a shoulder near 933.2 eV. This anomaly is only visible for the EuBCO and Eu_0.8_Pr_0.2_BCO superconductors, which is consistent with the oxygen K-edge results.

In addition, the Pr M4,5-edges enable the estimation of the Pr valence state (Figure 10b). A simple distinction between 3^+^ and 4^+^ is due to the peak at 946.5 eV, indicating a trivalent Pr^3+^ ion in the (Eu,Pr)BCO series.

In turn, the Eu M4,5-edges lose intensity in the XAS spectrum with the Pr content (Figure 10c). The Eu/Pr substitution effect is that all spectral lines are slightly shifted toward the higher energies, and similar to Pr, the Eu ion is trivalent.

On the other hand, the Ba M4,5-edges do not show any disturbances as a function of Pr content (Figure 10d), which means no significant changes occur in the Ba sublattice. Furthermore, only a tiny pre-peak at 781.4 eV, which corresponds to the forbidden ^3^*P*_1_ transition, indicates a small hybridization of the Ba 6*s*6*p* states with Cu 3*d* electrons.

Since the half-width at full maximum (HWFM) of the Ba M5 peaks are the same within the uncertainty levels, one may conclude that atomic mixing between the Eu/Ba and Pr/Ba sites is similar. Therefore, these results suggest that atomic disorder is not the main reason for superconductivity suppression in (Eu,Pr)BCO compounds.

### 3.5. Magnetization Measurements

**Figure 11:**
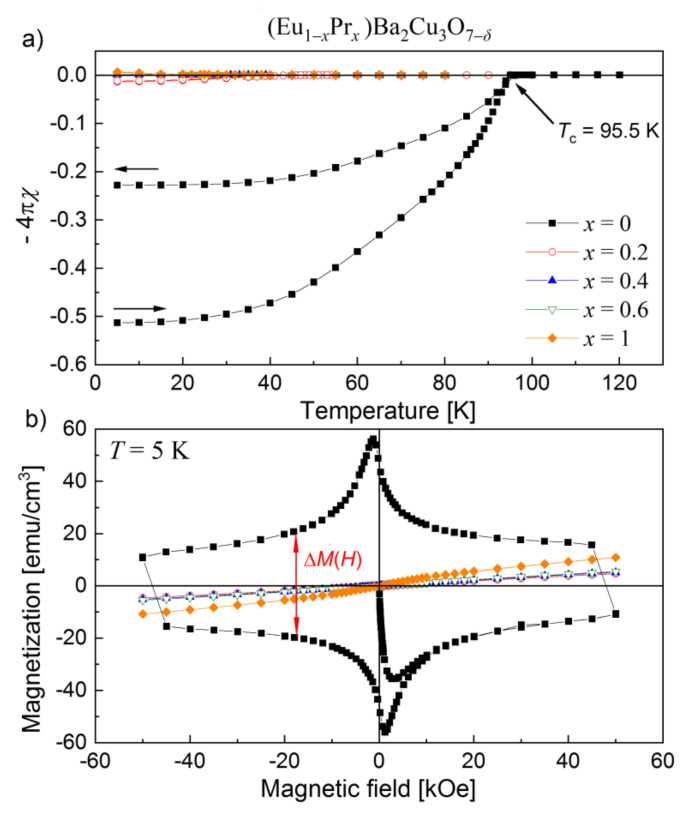
Magnetic curves for (Eu,Pr)BCO system: (**a**) DC magnetic susceptibilities, (**b**) hysteresis loops recorded at 5 K.

The magnetic susceptibility curves of (Eu,Pr)BCO are shown in Figure 11a, and the hysteresis loops recorded at 5 K are presented in Figure 11b.

EuBCO transition from the normal state to superconductivity is seen at 95.5 K (Figure 11a). By analyzing the magnetization data as *−*4π·*χ*(*T*), one can see how drastically the Pr atoms incorporated into the (Eu,Pr)BCO system reduce the Meissner effect. For Eu_0.8_Pr_0.2_BCO, the critical temperature (*T*_c_) is lowered twice to 45 K.

In addition, the complete disappearance of thermal hysteresis of the *M*(*T*) curves (Figure 11a) suggests a strong suppression of the superconducting properties induced by praseodymium. A related effect is also seen in Figure 11b, where there is a broad hysteresis loop for EuBCO, which is characteristic of superconductors. At the same time, for (Eu,Pr)BCO compounds, almost the straight *M*(*H*) lines typical for paramagnetic or antiferromagnetic materials are detected.

For *x* ≥ 0.4, the superconducting state is completely lost (positive *χ* in Figure 11a), which is not surprising, since many (RE,Pr)BCO compounds exhibit a Pr-induced suppression of the superconductivity [58,59,60,61,62,63,64,65,66,67,68,69,70,71].

Using the Bean critical-state model [72,73,74], it is possible to estimate the superconducting critical current density (*J_c_*) of (Eu,Pr)BCO according to the formula:

(2) $$J_{c}\left(H\right)=\frac{20\cdot \Delta M\left(H\right)}{a\cdot \left(1-\frac{a}{3b}\right)}$$

where *a* and *b* are the sample dimensions perpendicular to the applied magnetic field, and Δ*M* is a difference in magnetizations *M*_↓_–*M*_↑_ when sweeping the *H* field down and up, respectively.

**Figure 12:**
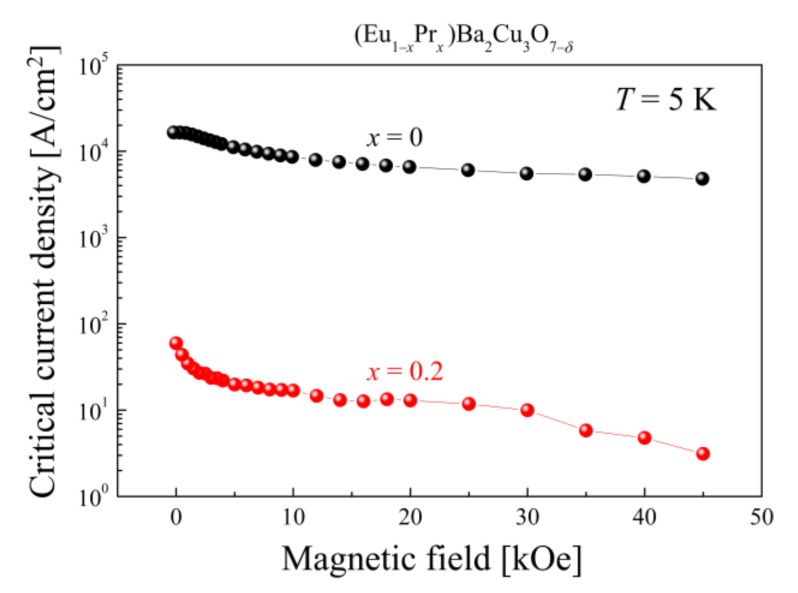
Critical current densities for EuBCO and Eu_0.8_Pr_0.2_BCO as a function of the magnetic field.

As mentioned above, estimating the critical current density only becomes meaningful for EuBCO and Eu_0.8_Pr_0.2_BCO. A significant *J*_c_ attenuation from two to three orders of magnitude for Eu_0.8_Pr_0.2_BCO is shown in Figure 12. There is also a slight loss of *J*_c_ along the internal magnetic field, confirming its destructive nature for the superconducting state.

### 3.6. Oxygen Index

The collected results consistently describe the properties of (Eu,Pr)BCO except for Eu_0.6_Pr_0.4_BCO, where the structural and spectroscopic measurements concerning magnetic studies exhibit some discrepancies. Furthermore, the Eu_0.6_Pr_0.4_BCO material is controversial as to the nature of conductivity, as not all investigation methods indicate its superconductivity state. Therefore, it was decided to resolve this uncertainty by determining the oxygen index by the iodometric titration method.

**Figure 13:**
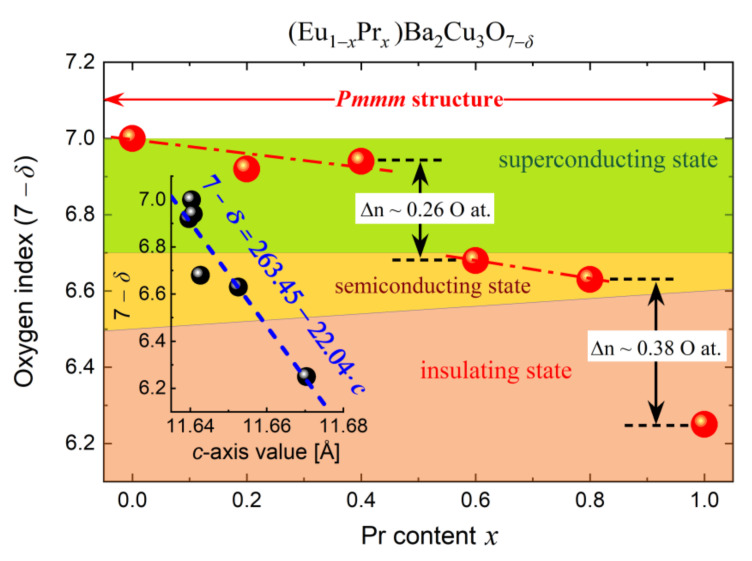
Schematic diagram of the conductivity regions for (Eu,Pr)BCO as a function of Pr content and *c*-axis value of *Pmmm* orthorhombic unit cell (inset).

The results are presented in Figure 13, where the gradual loss of oxygen in the *Pmmm* structure is visible. This phenomenon slowly progresses up to Eu_0.6_Pr_0.4_BCO; then, a rapid decrease in the oxygen content (Δ*n* = 0.24) is observed. The same rule applies to a further increase in the Pr content. The Eu_0.4_Pr_0.6_BCO and Eu_0.2_Pr_0.8_BCO samples show a similar O concentration, while PrBCO exhibits a further oxygen loss of Δ*n* = 0.38.

The above observations allowed the discrimination of three areas of different conductivity types. First, the most oxygenated samples up to *x* = 0.4 are characterized by the superconductivity state at low temperatures.

Second, samples with a higher Pr content lose their superconducting properties at a macro scale, passing into the ohmic conduction mechanism. However, this behavior does not exclude a mixed (vortex) state with small intragranular superconductivity and dominant ohmic conductivity. In contrast, PrBCO does not possess any superconducting properties at all.

Similar conclusions can be drawn from the dependence of the c lattice parameter on the oxygen index 7–*δ* (inset in Figure 13). Considering a strong negative correlation due to relation 7–*δ* = 263.45–22.04·*c*, the higher Pr content elongates the size of the unit cell in the [001] direction, which makes the *Pmmm* structure easier to adopt oxygen atoms in the Cu–O_2_ planes (reduction of holes) than in the metallic Cu–O_1−_*_δ_* layers responsible for the superconductivity state of the REBCO materials.

## 4. Summary

A solid-state reaction method was proven to be an effective procedure for synthesizing the (Eu,Pr)BCO materials. The sinters are characterized by dense, compact microstructures with slight inclusions of BaCuO_2_ and CuO.

According to XRD data, the orthorhombic *Pmmm* structure was the dominant phase for all (Eu,Pr)BCO. Furthermore, low-temperature XRD studies evidenced that phononic properties depend on the molar mass of Eu and Pr (strong dependence of the Debye temperature), while the actual lattice volume is a secondary factor.

For conventional superconductors, a linear electron–phonon coupling is the foundation of the BCS theory [75]. In this formalism, Debye temperature (*θ*_D_) growth means enhancing the electron–phonon coupling parameter (*λ*) due to the increase of phononic frequencies, which is reflected in the so-called isotopic effect.

However, for HTS materials, the role of electron–phonon interaction is not straightforward. Commonly, no isotopic effect is observed, which is often incorrectly attributed to a lack of electron–phonon coupling. In fact, the electron–phonon coupling in cuprates is much stronger and nonlinear than in conventional superconductors [76]. Therefore, for HTS materials, the electronic structure and lattice dynamics are crucial for discussing superconductivity suppression.

For cuprates, acoustic and optical phonons related to Cu–O bonds in- and out-of-plane are essential to establish the electron–phonon coupling [75,76]. Moreover, the acoustic phonons energies are enhanced for PrBCO compared to EuBCO, as evidenced by the rising Debye temperature. For this reason, it should be concluded that the electronic properties of (Eu,Pr)BCO and optical phonons are mainly responsible for HTS state suppression.

Indeed, XAS and Raman spectroscopies clearly showed a gradual deterioration of HTS state with Pr content driven by hole depletion of the Cu–O_2_ planes. The stretching mode Cu(1)–O(4) along the *c*-axis (500 cm^−1^) is known to be sensitive to oxygen content, and the lower oxygen concentration in the superconducting phase is expected to redshift this peak. However, the increase of praseodymium in the (Eu,Pr)BCO system splits a 500 cm^−1^ band into two lines, which is characteristic for the superconductivity state (485 cm^−1^) and ohmic conductivity (530 cm^−1^). This behavior reliably describes the gradual disappearance of the HTS state by suppressing the intragranular scale superconductivity for *x* > 0.4 due to insufficient oxygen factor.

On the other hand, XAS showed that for *x* > 0.4, the Cu 2*p*3*d*^10^L final state indicating high mobility of holes in the Cu–O_2_ planes is absent. Therefore, the depletion of the carriers and their localization is a leading mechanism of HTS loss in Eu_1−*x*_Pr*_x_*BCO. The above discussion is summarized in the form of a schematic diagram presented in Figure 13.

## Figures and Tables

**Table 1:** Stoichiometry of Eu and Pr as derived from EDS for Eu_1−*x*_Pr*_x_*BCO compounds. Presented results aggregate several randomly selected areas with dimensions of 60 × 60 µm^2^ (equivalent to the total area in Figure 2a).

Stoichiometric Parameter	Eu_0.8_Pr_0.2_BCO	Eu_0.6_Pr_0.4_BCO	Eu_0.4_Pr_0.6_BCO	Eu_0.2_Pr_0.8_BCO
*x*	0.20 ± 0.04	0.43 ± 0.06	0.64 ± 0.07	0.82 ± 0.06

**Table 2:** Weight fractions of isolated crystalline phases for (Eu,Pr)BCO as derived from Rietveld analysis.

Compound	(Eu,Pr)BCO [wt%]	BaCuO_2_ [wt%]	CuO [wt%]
EuBCO	96.0 ± 0.9	2.0 ± 0.2	2.0 ± 0.2
Eu_0.8_Pr_0.2_BCO	88.6 ± 0.9	4.2 ± 0.2	7.2 ± 0.3
Eu_0.6_Pr_0.4_BCO	92.1 ± 0.9	1.9 ± 0.2	6.0 ± 0.3
Eu_0.4_Pr_0.6_BCO	83.7 ± 0.7	8.2 ± 0.3	8.1 ± 0.4
Eu_0.2_Pr_0.8_BCO	83.4 ± 0.8	9.2 ± 0.5	7.4 ± 0.3
PrBCO	95.0 ± 0.9	2.0 ± 0.2	3.0 ± 0.2

**Table 3:** Debye temperatures of individual (Eu,Pr)BCO.

Compound	*V*_o_ [Å^3^]	*θ*_D_ [K]	*I*_C_ [Å^3^/K]
EuBCO	174.89 ± 0.04	351.5 ± 2.9	0.018 ± 0.003
(Eu_0.4_Pr_0.6_)BCO	175.35 ± 0.04	366.1 ± 3.9	0.020 ± 0.004
PrBCO	174.79 ± 0.03	386.4 ± 3.5	0.019 ± 0.003

## Data Availability

The data presented in this study are available on request from the corresponding author.
